# Enabling Recovery: The Principles and Practice of Rehabilitation Psychiatry (2nd edn)

**DOI:** 10.1192/pb.bp.115.052621

**Published:** 2016-12

**Authors:** Debbie Mountain

The first edition of Enabling Recovery was a much-welcomed arrival for the discipline of rehabilitation psychiatry as it emerged from its marginal ‘resettlement’ function in the late 1990s. The discipline is now accepted as part of mainstream psychiatric practice.

This second edition takes into account new data and developments, and adds an international perspective. The book covers a wide range of topics over 31 chapters, comprehensively written by 42 contributors from diverse backgrounds, including researchers, clinicians, patients, managers and policy makers. To start with, there are historical accounts of altered mental states and how cultures through the ages have understood them and protected/treated people. The authors integrate themes of recovery, personalisation, social exclusion and disability with contemporary practice. Interventions are described – medication, a range of psychological therapies, physical healthcare and guidance around challenging behaviour and substance misuse. The section discussing service delivery in a range of settings presents the rehabilitation pathway as a whole-system approach, moving through different settings as people become more independent. It usefully details what is required for effective in-patient care, community care and supported accommodation, housing and work. Also included are special topics such as brain injury, autism, risk management and international psychiatry and, finally, a chapter on the expanding evidence base for the practice of specialist rehabilitation.

Although the book focuses on rehabilitation psychiatry, its appeal is wider and it is useful to multidisciplinary mental health teams, medical students and other healthcare practitioners. It is easy to read – the diversity of topics allows each chapter to stand alone so readers can dip in and out of the book as they please. It also functions as a ‘toolkit’ to support shared decision-making when faced with complexity. The chapters ‘Assessment for Rehabilitation’ and ‘Rehabilitation at the Coalface’ are particularly handy as they describe practical approaches to developing formulations, engaging with people and developing goals and treatment plans.

